# Coping strategies as a causal mediator of the effect of loss-related memory characteristics and negative loss-related appraisals on symptoms of PGD, PTSD and depression

**DOI:** 10.1017/S0033291721003123

**Published:** 2023-03

**Authors:** Kirsten V. Smith, Anke Ehlers

**Affiliations:** 1 Department of Experimental Psychology, University of Oxford, Oxford, UK; 2 Oxford Health NHS Foundation Trust, Oxford, UK; 3 The Loss Foundation (Registered Charity 1147362), London, UK

**Keywords:** appraisals, bereavement, cognitive behavioural therapy, coping strategies, depression, grief, mediation, memory, PTSD, structural equation modelling

## Abstract

**Background:**

Psychological models of posttraumatic stress disorder (PTSD) and prolonged grief disorder (PGD) make predictions about the role of unhelpful coping strategies in maintaining difficulties by blocking self-correction of negative appraisals and memory integration following stressful life events like bereavement. However, few studies have tested these predictions directly.

**Method:**

We used counterfactually based causal mediation to assess whether unhelpful coping strategies mediated the relationship between (1) loss-related memory characteristics and/or (2) negative grief-related appraisals and symptoms of PGD, PTSD and depression using a three-wave longitudinal sample (*N* = 275). Appraisals and memory characteristics were measured at time point 1, unhelpful coping strategies at T2, and symptom variables at T3. Additionally, multiple mediation analyses within a structural equation modelling (SEM) framework assessed which types of coping strategies differentially mediated symptoms of PGD, PTSD and depression.

**Results:**

Coping strategies mediated the relationship between negative appraisals and memory characteristics and symptoms of PGD, PTSD and depression after adjusting for demographics and loss characteristics. Sensitivity analyses suggested that these results were most robust for PGD, followed by PTSD and then depression. Multiple mediation analyses suggested that all four subscales (avoidance, proximity seeking, loss rumination and injustice rumination) individually mediated the effect of memory characteristics and appraisals on PGD.

**Conclusions:**

These results suggest that core predictions of the cognitive model for PTSD and the cognitive behavioural model of PGD are useful in predicting symptoms of post-loss mental health problems in the first 12–18 months after loss. Targeting unhelpful coping strategies is likely to reduce symptoms of PGD, PTSD and depression.

## Introduction

Bereavement can lead to a range of mental health problems such as prolonged grief disorder (PGD), posttraumatic stress disorder (PTSD) and depression. Successful psychological therapies address these difficulties by targeting cognitive processes thought to be involved in the development and maintenance of each problem (Beck, 1979; Boelen, 2006; Ehlers & Clark, 2000; Shear et al., 2007). Of particular importance is the identification of modifiable maintenance factors of distress. These can be defined as variables that block the natural resolution of symptoms. Cognitive theory makes a theoretical prediction that unhelpful coping strategies prevent self-correction of negative appraisals (Beck, 1979). When negative appraisals about the self, world, future, and others form following a loss, associated avoidance of situations, places, or people prevents individuals from making positive experiences that have the potential to shift unhelpful beliefs (e.g. the appraisal that the future is hopeless without the deceased persists if new activities or previously enjoyed ones are avoided) (Shear et al., 2007).

Within Ehlers and Clark's (2000) cognitive model for PTSD, unhelpful coping strategies are hypothesised to maintain symptoms of PTSD by preventing change of excessively negative meanings of the trauma and its aftermath and by preventing the trauma memory from being updated with information that changes the currently threatening meaning of its worst moments (Ehlers & Clark, 2000; Ehlers, Hackmann, & Michael, 2004). More recently, psychological models of prolonged or complex grief propose a similar role for coping strategies in PGD (Boelen, van den Hout, & van den Bout, 2006; Shear et al., 2007). It is hypothesised that any failure to integrate new information about the loss within its context of other autobiographical memories will give rise to feelings of disbelief, confusion and yearning. Typically, such failures are predicted by the extent to which an individual actively blocks integration via unhelpful coping strategies such as avoidance of loss-related memories, thoughts and associated activities (Boelen et al., 2006; Shear et al., 2007). A number of observational longitudinal studies have demonstrated the mediating role of coping strategies on the relationship between predisposing factors such as attachment (Delespaux, Ryckebosch-Dayez, Heeren, & Zech, 2013), neuroticism (van der Houwen, Stroebe, Schut, Stroebe, & Bout, 2010), violent loss (Boelen, de Keijser, & Smid, 2015), loss-centrality (Boelen, 2012) and psychopathology following bereavement. These studies have helped elucidate how coping strategies are employed by different personality types with different loss circumstances and how these relationships influence symptoms of PGD. Similarly, randomised controlled trials have shown that reductions in avoidant coping mediate treatment effects to reduce symptoms of grief and depression symptoms when measured concurrently (Smith, Tarakeshwar, Hansen, Kochman, & Sikkema, 2009).

However, to our knowledge, no studies have yet utilised a bereaved sample to examine the core hypotheses of Ehlers and Clark's (2000) cognitive model of PTSD and Boelen et al.'s (2006) model of PGD, i.e. that difficulties in memory integration and negative appraisals lead to bereavement-related mental health problems such as PGD, PTSD and depression, and that these effects are mediated by unhelpful coping strategies.

One reason for this may be the lack of adequate measurement tools for the characteristics of memory that may be linked to loss integration. Previous research aimed at measuring whether PGD is associated with the extent to which a bereavement has been integrated into the autobiographical memory base has focused on overgeneral memory using the autobiographical memory task (Boelen, Huntjens, van Deursen, & van den Hout, 2010; Maccallum & Bryant, 2010) memory intrusiveness from a trauma questionnaire (Boelen, 2012; Halligan, Michael, Clark, & Ehlers, 2003), and a sense of unrealness regarding the loss (Boelen, 2010). Although these are important candidate processes in failures of loss integration, it is also possible that they represent only some of the memory characteristics that likely play a role. Here, we used the newly developed Oxford Grief-Memory Characteristics scale (OG-M) to measure the characteristics of loss memories, which we hypothesise may act as a metric for the extent to which a loss has been integrated within the context of other autobiographical memories (Ehlers, 2006). This scale includes different aspects of loss-related memories such as triggers, content, valence, physical, emotional and behavioural consequences, intrusiveness, unrealness and vividness.

Following the theoretical predictions of Boelen et al.'s (2006) cognitive behavioural model (CBT) of PGD and Ehlers and Clark's (2000) cognitive model of PTSD (Ehlers & Clark, 2000), we aimed to determine whether longitudinal data collected from the Oxford Grief Study were compatible with the hypothesis that the effects of loss-related memory characteristics and negative loss appraisals on symptoms of PGD and PTSD would be in part mediated by unhelpful strategies of coping with the loss. We aimed to determine the specificity of these predictions by investigating mediation models in their association with symptoms of depression, as a common comorbid difficulty observed alongside PGD and PTSD. Theoretical accounts of depression predict coping strategies mediates the relationship between negative appraisals about the self, world and others and symptoms of depression (Beck, 1979), however the relationship between memories and depression via coping remains unclear.

Furthermore, to aid specific clinical recommendations, we aimed to identify what types of coping strategies are important for symptoms of PGD, PTSD and depression by conducting multiple mediation analyses of the four coping strategies subscales (avoidance, proximity seeking, loss rumination and injustice rumination).

## Methods

### Participants

Participants were 275 adults (age; *M* = 46.43, s.d. = 13.24, 79% women) recruited between 0 and 6 months after bereavement (months since loss; *M* = 2.94, s.d. = 2.01, range 0–8 months) and followed up 6 and 12 months later. Nine percent of the sample had lost a loved one to violent means, 30% (*N* = 83) had lost a partner, 9% (*N* = 24) had lost a child, 38% (*N* = 105) had lost a parent, 6% (*N* = 16) had lost a sibling, 14% (*N* = 39) another relative and 3% (*N* = 8%) a close non-relative (see also Smith & Ehlers, 2020). All participants were recruited via the internet through bereavement charity email lists, social media advertisements and through keyword-based advertising on the Google content network.

### Procedure

Measures of loss-related memory characteristics, negative appraisals at T1 (0–6 months), unhelpful coping strategies at T2 (6–12 months) and measures of psychopathology at T3 (12–18 months) were completed online in accordance with ethical guidelines (Smith, Thew, & Graham, 2018).

### Measures

#### Symptom measures

##### Prolonged Grief Disorder Inventory (PG-13)

The PG-13 measures the PGD criteria proposed by Prigerson et al. (2009). It assesses the prevalence and intensity of PGD symptoms in the past month (e.g. yearning for the deceased, feelings of emotional numbness/detachment from others and feeling that a part of oneself died along with the deceased). A continuous PGD total score can be determined by summing the items. Internal consistency of the PG-13 was excellent (*α* = 0.92). This criterion has been cited as the closest conceptualisation to, and the empirical basis for, the newly developed ICD-11 and DSM-5-TR criteria for PGD (Killikelly & Maercker, 2018; Prigerson, Boelen, Xu, Smith, & Maciejewski, 2021).

##### Posttraumatic stress disorder checklist for DSM-5 (PCL-5)

The PCL-5 (Weathers et al., 2013) assesses distress associated with the 20 symptoms of PTSD specified in DSM-5 over the past month. Items are rated on a five-point scale, from 0 (not at all) to 4 (extremely) with respect to symptoms arising as a result of their bereavement. The PCL-5 demonstrated excellent internal consistency (*α* = 0.96).

##### Patient Health Questionnaire (PHQ-9)

The PHQ-9 (Kroenke, Spitzer, and Williams, 2001) is based on the Diagnostic and Statistical Manual, 4th Edition, Text Revision criteria for major depressive disorder (DSM-IV-TR, American Psychiatric Association, 2000). It mirrors the nine major depressive symptoms, with each item scored on a 0 (not at all) to 3 (nearly every day) scale in the last 2 weeks and demonstrated excellent internal consistency (*α* = 0.93).

#### Cognitive measures

The Oxford Grief measures were developed as a battery of cognitive and behavioural factors associated with the development and maintenance of PGD. Content development was informed by the literature on cognitive processes in PTSD (Foa, Ehlers, Clark, Tolin, & Orsillo, 1999; Halligan et al., 2003) and grief (Boelen & Lensvelt-Mulders, 2005; Eisma et al., 2014), the expertise of therapists working in traumatic bereavement, and through qualitative interviews with bereaved individuals experiencing typical and prolonged grief (Smith, Rankin, & Ehlers, 2020). These measures were all found to have good to excellent internal consistency, test–retest reliability and criterion and convergent validity (Smith & Ehlers, 2019). Exploratory and confirmatory factor analyses of the OG measures supported a unidimensional structure for the memory characteristics scale, a five factor structure for the negative appraisals scale and a four factor structure for the coping strategies scale (Smith & Ehlers, 2019).

##### Loss-related memory characteristics (OG-M)

Twenty-seven items probed memory triggers and their consequences (e.g. ‘*I am reminded of the loss for no apparent reason*’), qualities of memory (e.g. ‘*Memories of things we did together are painful*’), the physical impact of loss-related memories (e.g. ‘*The memories of* [*-*]*'s death make my body ache with overwhelming fatigue*’) and were rated on a 5-point scale (0 – not at all to 4 – very strongly) in the past month, as well as the frequency and qualities of unintentional memories of the loss. Internal consistency was excellent (*ω* = 0.97).

##### Negative grief appraisals (OG-A)

This 35-item questionnaire asks participants to indicate on a 7-point scale (1 – totally disagree to 7 – totally agree) the extent to which they agree with the statements in the past month. Items pertain to five content domains: (1) Loss of self and life – 10 items (e.g. ‘*Without* [*-*] *I can never be strong again*’), (2) Regret – 3 items (e.g. ‘*I blame myself for things I did or did not do when* [*-*] *was alive*’), (3) Catastrophic consequences of grief – 7 items (e.g. ‘*If I start to cry I won't be able to stop’*), (4) Loss of relationships and future – 9 items (e.g. ‘*I cannot maintain previous relationships without* [*-*]’) and (5) Fear of losing connection to the deceased – 6 items (e.g. ‘*If I don't do everything I can to feel close to* [*-*] *I will lose them forever*’). Internal consistency was excellent (*α* = 0.97).

##### Unhelpful coping strategies (OG-CS)

This 23-item questionnaire asks participants on a 5-point scale (1 – never to 5 – always) to indicate how often they used particular strategies to cope with their loss. Items pertain to four content domains: (1) Avoidance – 6 items (e.g. ‘*I avoid places we went together*’), (2) Proximity seeking – 7 items (e.g. ‘*I neglect other things because I spend a lot of time doing things for* [*-*] (e.g. *creating memorials, fundraising*’), (3) Loss rumination – 7 items (e.g. ‘*I dwell on moments that could have changed the outcome*’) and (4) Injustice rumination – 4 items (e.g. ‘*I think over and over about how it could be that this happened*’). Internal consistency was excellent (*α* = 0.93).

### Data analysis

Data were analysed using counterfactually based causal definitions of direct and indirect effects in Mplus (Version 7.4; Muthén & Muthén, 2007; Pearl, 2011). Using this method the pure natural direct effect (PNDE) and total natural indirect effect (TNIE) are extracted as the product of the total effect the independent variables on the outcome (Muthén & Asparouhov, 2015). The TNIE (indirect) reflects the effect of appraisals and memory characteristics on symptoms via unhelpful coping strategies. The PNDE (direct) is the effect of appraisals and memory characteristics on symptoms not accounted for by unhelpful coping strategies. Calculation of the PNDE and the TNIE allows us to make causal inferences about the impact of coping strategies as a mediator of post-loss mental health problems. These paths are calculated by specifying two levels of each predictor. In the case of continuous predictors the mean and the standard deviation of the latent variables is chosen (Muthén & Asparouhov, 2015). Causal mediation assumes that, conditional on covariates, there are no unmeasured confounders of the predictor–mediator, predictor–outcome and mediator–outcome relationships, also known as sequential ignorability (Forastiere, Mattei, & Ding, 2018). By estimating the direct and indirect effects in the presence of an increasing value of rho *ρ* (i.e. the correlation of the residuals of the mediator and outcome variables) one can estimate the extent to which the mediation results are likely to remain important when including unmeasured confounders (e.g. autocorrelations)^†^^1^ (Imai, Keele, & Yamamoto, 2010). Here, we report *ρ* as a measure of sensitivity when the lower confidence interval (CI) of the indirect effect includes 0 (i.e. becomes non-significant). These models estimated direct and indirect relationships of cognitive predictors simultaneously controlling for the shared variance between memory characteristics and appraisals.

**Fig. 1.**
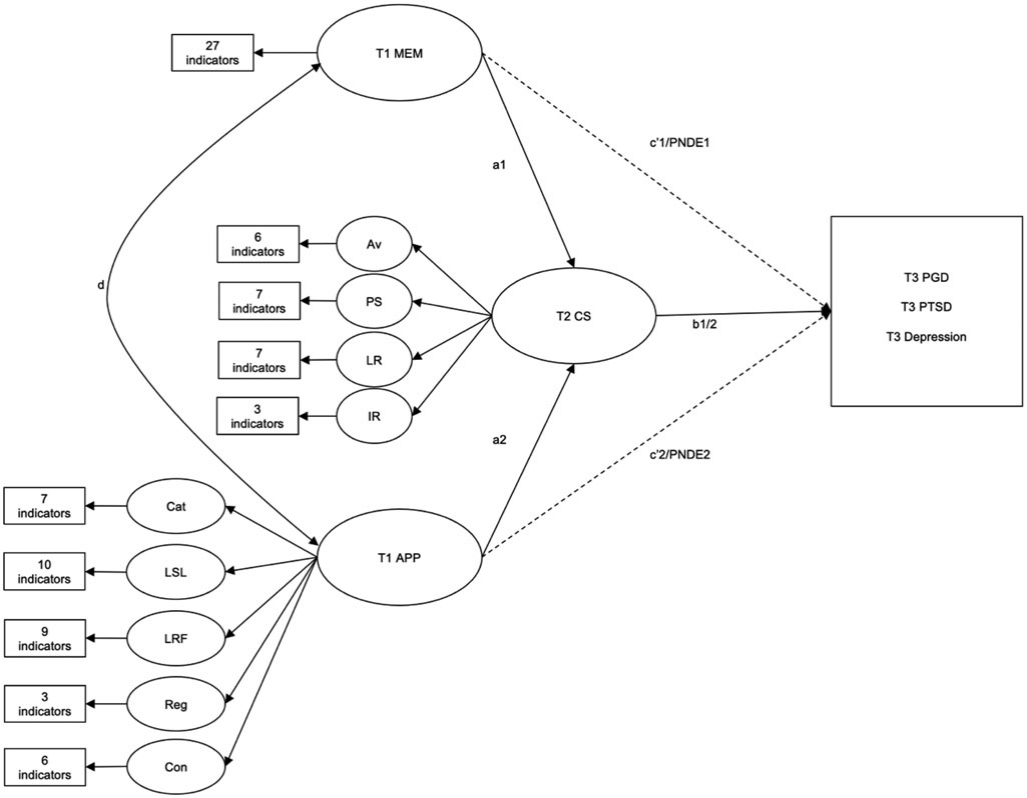
Causal mediation models for symptoms of PGD, PTSD and depression. *Note:* MEM, memory characteristics; APP, appraisals (Cat, catastrophic consequences of grief; LSL, loss of life and self; LFR, loss of future and relationships; Reg, regret; Con, fear of losing connection to the deceased); CS, coping strategies (Av, avoidance; PS, proximity seeking; LR, loss rumination; IR, injustice rumination); PGD, prolonged grief disorder; PTSD, posttraumatic stress disorder. Paths a1, b1, and PNDE1 correspond to the mediation model between memory characteristics, coping strategies and psychological outcome. Paths a2, b2 and PNDE2 correspond to the mediation model between appraisals, coping strategies and psychological outcome. Path d corresponds to the correlation between appraisals and memory characteristics at T1 which was included to model their shared variance. All models adjusted for gender, child loss, partner loss, violent loss, months since death and education.

The robust weighted least squares (WLSMV) extraction method suitable for outcomes with mixed distributions was used to evaluate hypotheses. As a precursor to mediation analyses, and to preserve the latent factor structure of the data, three confirmatory factor analyses were run to determine whether models specifying a relationship between memory characteristics, appraisals and coping strategies and PGD (model 1), PTSD (model 2) and depression (model 3), respectively, were an adequate fit to the data. Next, SEM was used to specify three mediation models (Fig. 1) in which coping strategies at T2 are specified as a mediator between appraisals and memory characteristics at T1 and symptom outcomes of PGD, PTSD and depression at T3. The PNDE and the TNIE are reported after adjustments for demographic variables and loss characteristics of importance. Smith and Ehlers (2020) found gender, level of education and type of loss – specifically losing a child (Yes/No) and losing a partner (Yes/No) to be associated with higher grief. Other research has reported months since loss and experience of a violent loss as predictors of grief severity (Lobb et al., 2010; Smith & Ehlers, 2021). Therefore, mediation analyses are reported after adjusting for gender, education, losing a child or a parent, violent loss and months since loss.

To further examine the direction of the mediated effect, a series of models were computed which were identical to those in Fig. 1 apart from the mediator and outcome variables, which were swapped. These, therefore, examined the ‘reverse’ mediation hypothesis, using PGD, PTSD or depression at T2 as the potential mediator, and coping strategies at T3 the dependent variable after adjusting for demographics and loss characteristics. These models allow a comparison of the influence of the outcome on the mediator, further adding to our understanding of causality.^2^

Determining the unique contribution of multiple parallel latent mediators in a counterfactual framework is not yet easily derived using Mplus (Muthén, Muthén, & Asparouhov, 2017). Therefore, we modelled six multiple mediation analyses (2 predictors × 3 symptom variables) in an SEM framework using the subscales of the OG-CS as parallel mediators and report the unique indirect effects for each mediator on symptoms of PGD, PTSD and depression.

Model adequacy was assessed by the χ^2^ goodness-of-fit test where the χ^2^:df ratio is smaller than 3:1 (Kline, 2015), comparative fit index (CFI) of 0.90 or higher as acceptable and 0.95 or higher as good, and root mean square error of approximation (RMSEA) of 0.08 or lower as acceptable and 0.06 as good (Hu & Bentler, 1999). Significance of paths in the mediation analyses was determined using 100 bootstrap resamples and examining bias-corrected CIs.

In order to investigate the influence of conceptual overlap between the characterisation of the cognitive mechanisms detailed in the questionnaires and the symptoms of PGD, PTSD and depression, 10 additional causal mediation models were analysed.^3^ Symptoms representing potential overlap (e.g. the avoidance item from the PGD conceptualisation) were systematically removed and reanalysed to determine the impact of potential confounding. The pattern of results reported below was unchanged.

## Results

### Confirmatory factor analyses

The confirmatory factor analyses (Table 1) showed adequate fit according to χ^2^ and CFI, and a good fit according to the RMSEA. All cognitive predictors were significantly associated with the psychological distress outcomes (*p* < 0.001).

**Table 1.** Goodness of fit indicators and predictor outcome estimates for CFA models

CFA model	χ^2^	df	χ^2/df^	CFI	RMSEA	Predictor estimate	Probable Dx criterion met
PGD (T3)	5159	3555	1.45	0.95	0.040		211 (8.7%)
T1 Appraisals						0.72***	
T1 Memory characteristics						0.61***	
T2 Coping strategies						0.81***	
PTSD (T3)	5166	3555	1.45	0.95	0.041		65 (26.5%)
T1 Appraisals						0.64***	
T1 Memory characteristics						0.56***	
T2 Coping strategies						0.71***	
Depression (T3)	5148	3555	1.45	0.95	0.040		88 (36.7%)
T1 Appraisals						0.53***	
T1 Memory characteristics						0.51***	
T2 Coping strategies						0.60***	

*Note:* Predictor estimates are correlations between predictors (memory characteristics, appraisals and coping strategies) and psychological symptoms (PGD, PTSD and depression).

T1 = time point 1 (0–6 months), T2 (6–12 months), T3 (12–18 months). Dx = diagnosis.

*** *p* < 0.001.

### Causal mediation analyses

Standardised path coefficients, bias-corrected bootstrapped CIs, total variance accounted for in psychopathology by the mediation model, sensitivity metric *ρ* and percentage of the variance accounted for by the indirect path via coping strategies are reported in Table 2. All three adjusted mediation models were an acceptable fit to the data.

**Table 2.** Model fit statistics, standardised path coefficients and causally mediated direct and indirect effect estimates for the PGD, PTSD, and depression-adjusted mediation models

									Covariates					
Model	Path	Coefficient	s.e.	Lower CI	Upper CI	*P*_M_	*ρ*	*R*^2^	1	2	3	4	5	6
PGD^a^								0.69	0.19***	0.39***	0.29***	−0.06	0.01	−0.02
Memory characteristics	a1	0.32***	0.09	0.12	0.46									
	b1	0.61***	0.09	0.41	0.82									
	PNDE 1	−0.13	0.10	−0.34	0.05									
	TNIE1	0.12**	0.04	0.07	0.21	48.00	0.40							
Appraisals	a2	0.54***	0.09	0.33	0.72									
	b2	0.61***	0.09	0.41	0.82									
	PNDE 2	0.19	0.10	−0.00	0.37									
	TNIE2	0.20***	0.05	0.10	0.31	51.28	0.40							
PTSD^b^								0.54	0.19***	0.32***	0.18*	−0.05	−0.08	−0.05
Memory characteristics	a1	0.32***	0.09	0.12	0.46									
	b1	0.52***	0.14	0.24	0.79									
	PNDE 1	−0.13	0.14	−0.38	0.17									
	TNIE1	0.10*	0.04	0.04	0.20	53.48	0.20							
Appraisals	a2	0.54***	0.09	0.33	0.72									
	b1	0.52***	0.14	0.24	0.79									
	PNDE 2	0.24	0.14	−0.02	0.52									
	TNIE2	0.17**	0.05	0.06	0.25	50.00	0.20							
Depression^c^								0.39	0.15**	0.31***	0.18*	−0.11	−0.06	−0.07
Memory characteristics	a1	0.32***	0.09	0.12	0.46									
	b1	0.39**	0.15	0.13	0.63									
	PNDE 1	0.08	0.12	−0.14	0.29									
	TNIE1	0.07**	0.04	0.02	0.16	46.67	0.10							
Appraisals	a2	0.54***	0.09	0.33	0.72									
	b2	0.39**	0.15	0.13	0.63									
	PNDE 2	0.04	0.14	−0.27	0.24									
	TNIE2	0.13**	0.05	0.04	0.21	76.47	0.10							

*P*_M_, percent mediation (i.e. the percentage of the total effect the predictor that is accounted for by the mediated path ab). *ρ* is the value of the correlated residuals of the mediator and outcome resulting from unmeasured confounders necessary to result in non-significance of the indirect effect. Covariates: 1 = gender, 2 = child loss, 3 = partner loss, 4 = education, 5 = months since death, 6 = violent loss.

*Note.* Path a represents the effect of the predictor (memory characteristics or appraisals) on the mediator variable (coping strategies). Path b represents the effect of the coping strategies on psychological symptoms PGD, PTSD and depression. PNDE represents the direct effect of the predictor on psychological distress controlling for the effect of coping strategies and demographic and loss characteristics. TNIE represents the indirect effect of the predictor on the symptom variables via unhelpful coping strategies. Coefficients are standardised. CIs are 95%.

a χ^2^ = 6228, df = 4065, χ^2/df^ = 1.53, CFI = 0.93, RMSEA = 0.044.

b χ^2^ = 6236, df = 4065, χ^2/df^ = 1.53, CFI = 0.93, RMSEA = 0.044.

c χ^2^ = 6219, df = 4065, χ^2/df^ = 1.53, CFI = 0.93, RMSEA = 0.044.

*p < 0.05, **p < 0.01, ***p < 0.001.

#### Prolonged grief disorder

The adjusted mediation model accounted for 69% of the variance in PGD. Bias-corrected bootstrapped CIs for the true natural indirect effects (memory characteristics: TNIE1 = 0.12, 95% CI 0.06–0.21; appraisals: TNIE2 = 0.20, 95% CI 0.10–0.31) based on 100 bootstrap samples were entirely above zero indicating that the paths from memory characteristics and appraisals to PGD were mediated by coping strategies. For both predictors, the PNDEs were not significant indicating that memory characteristics and appraisals at time point 1 do not lead to symptoms of PGD a year later via any other mechanism than coping strategies at time point 2. These indirect effects accounted for 48% and 51% of the effect of memory characteristics and appraisals on PGD respectively. The sensitivity analysis suggested that these relationships remained significant as long as *ρ* ⩽ about 0.4, indicating moderate sensitivity to violations of the sequential ignorability assumption.

We tested the reverse mediation model, where PGD at T2 was entered as the mediator and coping strategies at T3 as the outcome. The true natural indirect effects for memory characteristics and appraisals on coping via PGD symptoms were significant (memory characteristics: TNIE1 = 0.08, 95% CI 0.05–0.14; appraisals: TNIE2 = 0.08, 95% CI 0.05–0.15). The PNDEs of memory characteristics on coping strategies was significant (PNDE = 0.24, 95% CI 0.08–0.41) but the direct effect of appraisals was not (PNDE = 0.20, 95% CI −0.04 to 0.35). Sensitivity analyses were not available for reversed models as the outcome variable is latent.

#### Posttraumatic stress disorder

As with PGD both the true natural indirect effects for memory characteristics and appraisals, adjusted for demographics and loss characteristics, were significant according to the bootstrapped CIs (memory characteristics: TNIE1 = 0.10, 95% CI 0.04–0.20; appraisals: TNIE2 = 0.17, 95% CI 0.06–0.25). Direct effects were not significant after controlling for the impact of coping strategies. The total model accounted for 54% of the variance in PTSD symptoms and indirect effects accounted for 50% and 43% of the total effect of appraisals and memory characteristics on PTSD, respectively. The indirect effects remained significant up to *ρ* = 0.20.

In the reverse-adjusted mediation model the TNIEs were significant for appraisals (TNIE = 0.11, 95% CI 0.05–0.19) but not memory characteristics (TNIE = 0.05, 95% CI −0.02 to 0.10). Although the PNDE of memory characteristics on coping strategies was significant (PNDE = 0.28, 95% CI 0.15–0.47) but the PNDE of appraisal on coping strategies was not (PNDE = 0.16, 95% CI −0.15 to 0.29).

#### Depression

The indirect paths of appraisals and memory characteristics on symptoms of depression via coping strategies were significant (memory characteristics: TNIE1 = 0.07, 95% CI 0.02–0.16; appraisals: TNIE2 = 0.13, 95% CI 0.04–0.22). Direct effects were not significant. Indirect effects accounted for 47% of the effect of memory characteristics on depression and 76% of the effect of appraisals on depression. The total model explained 38% of the variance in depression. The value of *ρ* = 0.10 indicated that the model was highly sensitive to violations of the sequential ignorability hypothesis suggesting that unmeasured confounders may affect the results of the model.

The reverse mediation model indicated that only the TNIE for appraisals via depression was significant (memory characteristics: TNIE1 = 0.02, 95% CI −0.01 to 0.05; appraisals: TNIE2 = 0.04, 95% CI 0.02–0.08). The direct effects were significant (memory characteristics: PNDE1 = 0.33, 95% CI 0.15–0.49; appraisals: PNDE2 = 0.26, 95% CI 0.05–0.41).

### Investigating conceptual overlap

In all 10 additional causal mediation models significant indirect effects persisted with comparable levels of *ρ* (0.40–0.49 for PGD, 0.20–0.35 for PTSD, 0.10 for depression). All direct paths were non-significant as above.

### Multiple mediation

Standardised path coefficients and bias-corrected bootstrapped CIs are reported in Table 3. All six models were an acceptable fit to the data. All four subscales (avoidance, proximity seeking, loss rumination and injustice rumination) mediated the relationship of both memory characteristics and appraisals on symptoms of PGD. The direct effect of appraisals on PGD was not significant. However, the direct effect of memory characteristics on PGD was significant and negative, indicating that once unhelpful coping is accounted for by the model the remaining effect of memory characteristics predicted lower grief symptoms. When considering trauma symptoms, avoidance, proximity seeking and loss rumination significantly mediated the effect of memory characteristics on PTSD, while only avoidance and loss rumination mediated the effect of appraisals on PTSD. The direct effect of memory characteristics on PTSD was significant and negative, as with PGD, while the direct effect of appraisals was not significant. Finally, avoidance and loss rumination significantly mediated the effect of both memory characteristics and appraisals on depressive symptoms. The direct effects in the depression models were not significant.

**Table 3.** Model fit statistics, standardised path coefficients and direct and indirect effect estimates for the PGD, PTSD and depression mediation models

Coping strategies subscale		Memory characteristics						Appraisals					
		PGD^a^		PTSD^b^		DEP^c^		PGD^d^		PTSD^e^		DEP^f^	
		Est.	CI	Est.	CI	Est.	CI	Est.	CI	Est.	CI	Est.	CI
	DE	−1.37**	−2.03 to −0.60	−0.99*	−0.19 to −0.18	0.48	−1.28 to 0.89	−0.52	−0.99 to 0.04	−0.29	−0.94 to 0.72	−0.32	−1.38 to 0.40
Avoidance	IndE	0.49**	0.20–0.811	0.58**	0.36–1.06	0.37*	0.13–0.79	0.27*	0.10–0.48	0.41**	0.21–0.71	0.34*	0.07–0.65
Proximity seeking	IndE	0.64***	0.29–0.93	0.39*	0.11–0.78	0.23	−0.11 to 0.51	0.43**	0.15–0.69	0.19	−0.23 to 0.43	0.17	−0.20 to 0.55
Loss rumination	IndE	0.34**	0.18–0.59	0.33**	0.16–0.56	0.22*	0.04–0.43	0.22**	0.13–0.40	0.23**	0.07–0.36	0.20*	0.06–0.36
Injustice rumination	IndE	0.53***	0.20–0.74	0.26	−0.06 to 0.50	0.17	−0.08 to 0.38	0.32**	0.00–0.48	0.10	−0.33 to 0.26	0.14	−0.12 to 0.37

Est., estimate; CI, 95% confidence intervals; DE, direct effect; IndE, indirect effect.

Coefficients are standardised.

a χ^2^ = 2093, df = 1216, χ^2/df^ = 1.72, CFI = 0.95, RMSEA = 0.051.

b χ^2^ = 2100, df = 1216, χ^2/df^ = 1.73, CFI = 0.95, RMSEA = 0.051.

c χ^2^ = 2086, df = 1216, χ^2/df^ = 1.72, CFI = 0.95, RMSEA = 0.051.

d χ^2^ = 3015, df = 1639, χ^2/df^ = 1.84, CFI = 0.95, RMSEA = 0.055.

e χ^2^ = 3008, df = 1639, χ^2/df^ = 1.83, CFI = 0.95, RMSEA = 0.055.

f χ^2^ = 3005, df = 1639, χ^2/df^ = 1.83, CFI = 0.95, RMSEA = 0.055.

*p < 0.05, **p < 0.01, ***p < 0.001.

## Discussion

The current study used counterfactually defined causal effects to investigate the core cognitive processes (loss-related memory characteristics, negative appraisals and maladaptive coping strategies) in Ehlers and Clark's (2000) cognitive model for PTSD and Boelen et al.'s CBT model for PGD in their prediction of symptoms of PGD and PTSD in a longitudinal community sample of bereaved individuals. The loss-related cognitive predictors were also tested for their utility in predicting depression, as a common comorbidity following loss.

Coping strategies mediated the relationship between negative appraisals and PGD, PTSD and depression and the relationship between memory characteristics and PGD, PTSD and depression. These results fit with predictions from both Boelen et al.'s (2006) CBT of PGD, Ehlers and Clark's (2000) cognitive model for PTSD, and previous research that found therapies targeting unhelpful coping strategies such as avoidance through exposure to avoided emotions or stimuli were effective at reducing symptoms of PGD, PTSD and depression (Bryant et al., 2014; Eisma et al., 2015; Papa, Sewell, Garrison-Diehn, & Rummel, 2013). The direct effects for each model were not significant after controlling for the effect of unhelpful coping strategies. This suggests that the coping strategies at 6 months measured here fully capture the impact of initial appraisals and memory characteristics at T1 on symptoms of PGD, PTSD and depression 12 months later. Analyses were adjusted for demographics and loss characteristics previously shown to be related to symptoms of PGD, but the existence of other significant contributors cannot be ruled out. Therefore, we conducted sensitivity analyses to understand the extent to which these results would persist in the presence of unmeasured confounders.

The sensitivity analyses indicate that these results are the most robust for PGD, with PTSD and depression demonstrating high sensitivity to unmeasured confounders. This is perhaps not surprising given that the measures utilised in this study were designed to measure appraisals, memory characteristics and coping strategies typical of people with PGD, as opposed to depression and PTSD, which may have contributed to the lower sensitivity in these analyses. However, despite being designed specifically for grief reactions, the results show that the Oxford Grief measures are helpful in understanding symptoms of PTSD and depression and therefore may be useful in predicting a variety of symptoms following bereavement.

All three reverse mediation models showed indirect effects of reduced magnitude compared with the forwards mediation models. Although indirect effects of memory characteristics and appraisals were significant via PGD, only appraisals predicted coping strategies via PTSD and depression. These results support the causal relationships defined in the theoretical models of PGD (Boelen et al., 2006) and PTSD (Ehlers & Clark, 2000). The forwards mediation supports the development and maintenance of symptoms via cognitive processes while the reverse mediation points to a dynamic feedback loop, or vicious cycle, whereby symptoms influence the effect of cognitive variables on coping strategies. For example, believing that meaningful relationships are impossible without the deceased, may trigger intense emotional pain, prompting rumination about how the death could have been prevented.

In order to make some specific clinical predictions about the use of unhelpful coping following bereavement we used multiple mediation analyses to determine whether the subscales of the OG-CS differentially predicted symptoms of PGD, PTSD and depression. The results indicate avoidance, proximity seeking, loss rumination and injustice rumination all significantly mediated of the effect of memory characteristics and appraisals on PGD. This supports the utility of the individual subscales of the OG-CS as a clinical tool for guiding treatment of PGD by indicating the most pressing targets for treatment and in which order they should be addressed. For bereaved individuals suffering with trauma symptoms a specific focus on strategies of avoidance, proximity seeking and loss rumination may be more appropriate, whereas depressed individuals may benefit most from reducing strategies of avoidance and loss rumination.

Several limitations of the current study should be noted. Due to sample size constraints not all available data could be modelled in these analyses. Further studies with larger samples may be able to model cognitive mechanisms and symptoms at all time points in order to elucidate the role of potential unmeasured confounders such as autocorrelations. Given the strong relationships between cognitive predictors, likely inflated by high levels of resilience in our sample, validating these findings further in a clinical sample will be of particular importance. These analyses assess symptoms of PGD using the Prigerson et al. (2009) criterion. Several sets of diagnostic criteria for severe and enduring grief exist within the research literature and research has shown prevalence rates and predictive validity may differ by conceptualisation (Cozza et al., 2016; Maciejewski, Maercker, Boelen, & Prigerson, 2016). Recent guidance has suggested that clinical interviews covering multiple criteria sets may be helpful in creating consensus (Lenferink, Boelen, Smid, & Paap, 2019). The current study relied on self-report measures to assess the predictors, mediators and outcomes. Future research that utilises clinician administered assessments will likely be helpful in supporting the validity of these findings. Study participants were predominantly Caucasian. As such, the observed patterns may not generalise to other populations. Future studies would benefit from confirming these results in different cultural contexts. Furthermore, confirming the multiple mediation results within a causal framework when possible will add further tests of these findings. The measures we used were designed to measure appraisals and memory characteristics typical of people with PGD, and we did not assess depression- and PTSD-specific memory characteristics or appraisals, which may have contributed to the lower variance explained in these models (PTSD: *R*^2^ = 54; depression: *R*^2^ = 39) compared to those for PGD (*R*^2^ = 69). However, despite being designed specifically for grief reactions, the results show that the Oxford Grief measures predict as much variance in PTSD following loss as a previous study using a general trauma sample and PTSD specific measures of memory characteristics, appraisals and coping strategies (Beierl, Böllinghaus, Clark, Glucksman, & Ehlers, 2020).

Finally, the multiple mediation analyses were conducted using conventional direct and indirect effect within an SEM framework. Implementation of counterfactually based parallel multiple mediation analyses is not yet easily derived in Mplus. Given the second-order latent variable model using counterfactually defined effects indicated significant mediation for PGD, PTSD and depression we felt confident in the validity of moving to an SEM framework for our multiple mediation analyses. However, assessment of multiple mediators using sensitivity analyses will be helpful in future studies to confirm the generalisability of these results.

Notwithstanding these limitations, the current study has important clinical implications. Given that memory characteristics, appraisals and coping strategies are modifiable, they are appropriate targets for interventions with bereaved individuals seeking help. In addition, our finding that the relationship between memory characteristics, appraisals and PGD, PTSD and depression was fully mediated by unhelpful coping suggests that interventions aimed at reducing unhelpful strategies, and developing healthy coping, such as behavioural activation (Papa et al., 2013), ‘then *v.* now’ discrimination of memory triggers (Ehlers & Clark, 2000), exposure to avoided memories (Bryant et al., 2014; Shear et al., 2014) or stimuli (Eisma et al., 2015) may be particularly helpful in reducing trauma symptoms following bereavement. Clinicians offering bereavement support may want to consider initial levels of loss-related memory characteristics, and appraisals when developing a treatment plan and assess unhelpful coping to promote successful treatment implementation.

## Footnotes

† The notes appear after the main text.

## Notes

1 See online Supplementary Appendix A1.

2 Forwards and reverse mediation results without covariates can be found in online Supplementary Appendix A2 and show the same pattern as the analyses with covariates presented in the paper.

3 See online Supplementary Appendixes A3–A5.

## Supplementary material

For supplementary material accompanying this paper visit https://doi.org/10.1017/S0033291721003123. click here to view supplementary material
