# Supplementary material for: Coping strategies as a causal mediator of the effect of loss-related memory characteristics and negative loss-related appraisals on symptoms of PGD, PTSD and depression
Source: Psychol Med. 2021 Aug 27;53(4):1542–51. doi: 10.1017/S0033291721003123 (PMC10009377; doi:10.1017/S0033291721003123)
Supplement: Supplementary file 1 [file S0033291721003123sup001.pdf]

Table A1

Synchronous and asynchronous correlations of cognitive measures and symptom outcomes

Variable	1	2	3	4	5	6	7	8	9	10	11	12	13	14	15	16	17
1. T1 A																	
2. T1 CS	.77**																
3. T1 M	.79**	.83**															
4. T2 A	.86**	.74**	.73**														
5. T2 CS	.76**	.85**	.76**	.83**													
6. T2 M	.72**	.78**	.85**	.84**	.86**												
7. T3 A	.79**	.68**	.67**	.88**	.78**	.75**											
8. T3 CS	.68**	.79**	.71**	.77**	.85**	.80**	.82**										
9. T3 M	.69**	.75**	.77**	.78**	.80**	.86**	.83**	.88**									
10. T1 PGD	.79**	.82**	.86**	.72**	.78**	.80**	.68**	.71**	.72**								
11. T1 PTSD	.68**	.71**	.75**	.67**	.65**	.68**	.57**	.61**	.65**	.70**							
12. T1 DEP	.67**	.64**	.69**	.67**	.62**	.64**	.56**	.55**	.58**	.69**	.80**						
13. T2 PGD	.71**	.73**	.76**	.82**	.84**	.88**	.73**	.77**	.79**	.82**	.59**	.61**					
14. T2 PTSD	.66**	.68**	.69**	.79**	.78**	.83**	.69**	.73**	.76**	.70**	.76**	.69**	.80**				
15. T2 DEP	.55**	.55**	.56**	.71**	.63**	.69**	.55**	.58**	.59**	.55**	.58**	.65**	.69**	.79**			
16. T3 PGD	.71**	.70**	.71**	.79**	.80**	.82**	.85**	.82**	.87**	.76**	.56**	.54**	.85**	.76**	.61**		
17. T3 PTSD	.62**	.60**	.64**	.72**	.69**	.73**	.76**	.76**	.83**	.65**	.69**	.65**	.71**	.83**	.67**	.82**	
18. T3 DEP	.52**	.50**	.55**	.63**	.58**	.62**	.64**	.65**	.70**	.53**	.56**	.63**	.62**	.68**	.70**	.69**	.85**

Note. T1 = Time point 1, T2 = Time point 2, T3 = Time point 3, A = Appraisals, CS = Coping Strategies, M = Memory characteristics, PGD = Prolonged Grief Disorder (PG-13), PTSD = Posttraumatic Stress Disorder (PCL-5), DEP = Depression (PHQ)
** indicates $p < .01$.

Table A2

Comparison of forwards and reverse mediation analyses without covariates

Cognitive variables	Path	PGD				PTSD				Depression			
		Forward[a]		Reverse[b]		Forward[a]		Reverse[b]		Forward[a]		Reverse[b]	
		Est.	CI	Est.	CI	Est.	CI	Est.	CI	Est.	CI	Est.	CI
Memory characteristics	a1	.32***	.16 - .46	.31***	.11 - .48	.32***	.16 - 46	.28	-.12 - .44	.33***	.16 - .47	.17	-.07 - .34
	b1	.82***	.53 - 1.02	.45***	.30 - .56	.67***	.22 - .99	.39***	.24 - .51	.53*	.22 - .94	.23***	.09 - .33
	PNDE 1	-.22	-.46 - .02	.27*	.11 - .45	-.12	-.45 - .10	.31***	.10 - .46	.04	-.27 - .26	.37***	.20 - .57
	TNIE 1	.16**	.06 - .25	.08**	.04 - .16	.13*	.04 - .25	.06	.00 - .14	.10**	.04 - .22	.02	-.01 - .06
Appraisals	a1	.59***	.38 - .75	.47***	.31 - .61	.59***	.38 - .75	.45***	.22 - .70	.59***	.38 - .75	.42***	.24 - .60
	b1	.82***	.53 - 1.02	.45***	.30 - .56	.67***	.22 - .99	.39***	.24 - .51	.53*	.22 - .94	.23***	.09 - .33
	PNDE 1	.20	-.01 - .41	.22	-.01 - .40	.17	-.07 - .58	.26*	-.07 - .39	.04	-.33 - .28	.34**	.03 - .50
	TNIE 1	.29***	.15 - .39	.13***	.08 - .20	.24**	.10 - .35	.11**	.07 - .21	.19**	.08 - .33	.06**	.03 - .11

Note. Est. = Estimate. CI = 95% Confidence intervals. Coefficients are standardized. [a] Cognitive variables at T1 predict symptoms at T3 via coping strategies at T2.
[b] Cognitive variables at T1 predict coping strategies at T3 via symptoms at T2.
* $p < .05$ ** $p < .01$ *** $p < .001$

Investigating conceptual overlap

PGD.

Model 1: Items 7, 11, 13 of the PGD conceptualisation represent feelings regarding confusion about one's role in life, difficulty moving on or making plans for the future, and a sense that life is unfulfilling or meaningless since the loss, respectively. These symptoms conceptually overlap with the appraisals factors – Loss of self and life and Loss of relationships and future. Therefore, items 7, 11, and 13 were removed from the PGD total score and a causal mediation analysis of only appraisals as the predictor was run.

Models 2 and 3: Item 5 of the PGD scale measures avoidance of reminders that the deceased person is gone. The avoidance subscale of the coping strategies measure specifies specific activities avoided by the bereaved without an associated reason for avoidance but may represent conceptual overlap. Therefore, item 5 was removed and reanalysed for appraisals (model 2) and memory characteristics (model 3) separately.

Model 4: Removes the 4 symptom items described above and runs the mediation model including both appraisals and memory characteristics.

PTSD.

Model 5: Items 9 and 10 of the PTSD conceptualisation measure strong negative beliefs about the self, the world and others and blaming others or yourself for the loss. They overlap with the appraisals factors Loss of self and life and Regret and as such were removed from model 5 in which only appraisals was run.

Model 6: Items 1, 3, 4 of the PTSD scale measures intrusive memories, reliving of the stressful event, and feeling upset when reminded of the loss. The memory scale measures unwanted memories of the loss, the extent to which the memory was being relived in the here and now, and fatigue in response to memories. Model 6 removed items 1, 3, 4 of the PTSD scale and reran the mediation model with only memory characteristics.

Model 7 and 8: Items 6 and 7 of the PTSD scale measures avoidance of thoughts and feelings and avoidance of external reminders of the loss which overlaps with the avoidance subscale of the coping strategies measure. Models 7 and 8 removed this item from the PTSD scale and reanalysed appraisals (model 7) and memory characteristics (model 8) separately.

Model 9: Removes the 7 symptom items described above and runs the mediation model including both appraisals and memory characteristics.

Depression.

Model 10: Item 6 from the depression scale measures feeling bad about yourself or that you are a failure who has let your family down. This item overlaps with content in the Loss of self and life subscale of the appraisals measure. Therefore, item 6 was removed from the depression total and analyses repeated using only appraisals as a predictor.

Table A3

Mediation models removing overlapping PGD symptoms

									Covariates					
Model		Path	Coefficient	SE	Lower CI	Upper CI	ρ	R^2	1	2	3	4	5	6
	**PGD**													
1	Appraisals	a1	$.80^{***}$	.04	.73	.89		.68	$.19^{***}$	$.41^{***}$	$.26^{***}$	-.07	.01	.00
		b1	$.61^{***}$	.09	.40	.74								
		PNDE 1	.06	.09	-.08	.27								
		TNIE 1	$.30^{***}$	.05	.19	.37	.40							
2	Appraisals	a1	$.80^{***}$	.04	.73	.89		.68	$.19^{***}$	$.39^{***}$	$.29^{***}$	-.05	.02	-.02
		b1	$.57^{***}$	.09	.40	.75								
		PNDE 1	.12	.09	-.06	.32								
		TNIE 1	$.28^{***}$	.05	.18	.38	.40							
3	Memory characteristics	a1	$.77^{***}$	.04	.67	.82		.68	$.19^{***}$	$.39^{***}$	$.29^{***}$	-.05	.02	-.02
		b1	$.68^{***}$	.09	.51	.87								
		PNDE 1	-.03	.09	-.22	.13								
		TNIE 1	$.31^{***}$	.05	.23	.42	.49							
4	Appraisals	a1	$.54^{***}$	.09	.33	.72		.67	$.20^{**}$	$.41^{***}$	$.26^{***}$	-.05	.02	.00
		b1	$.59^{***}$	.10	.34	.75								
		PNDE 1	.01	.11	-.16	.21								
		TNIE 1	$.20^{***}$	.05	.08	.31	.40							
	Memory characteristics	a2	$.32^{***}$	.09	.12	.46								
		b2	$.59^{***}$	.10	.34	.75								
		PNDE 2	.08	.09	-.10	.21								
		TNIE 2	$.11^{**}$	.04	.05	.20	.40							

Note. In models 1 and 2 path a represents the effect of the predictor (appraisals) on the mediator variable (coping strategies). In model 3 path a path a represents the effect of the predictor (memory characteristics) on the mediator variable (coping strategies). In model 4 path a represents the effect of the predictors (appraisals and memory characteristics) on the mediator variable (coping strategies). Path b in all models represents the effect of the coping strategies on psychological symptoms PGD. PNDE represents the direct effect of the predictor on psychological distress controlling for the effect of coping strategies and demographic and loss characteristics. TNIE represents the indirect effect of the predictor on the symptom variables via unhelpful coping strategies. Coefficients are standardized. Confidence intervals are 95%.
ρ is the value of the correlated residuals of the mediator and outcome resulting from unmeasured confounders necessary to result in non-significance of the indirect effect.
Covariates 1 = Gender, 2 = Child loss, 3 = Partner loss, 4 = Education, 5 = Months since death, 6 = Violent loss.

Table A4

Mediation models removing overlapping PTSD symptoms

Model		Path	Coefficient	SE	Lower CI	Upper CI	ρ	R^2	Covariates 1	2	3	4	5	6
	**PTSD**													
5	Appraisals	a1	.80***	.04	.73	.89		.54	.19***	.32***	.21**	-.05	-.08	-.06
		b1	.49***	.12	.20	.67								
		PNDE 1	.15	.12	-.07	.39								
		TNIE 1	.24***	.06	.10	.33	.20							
6	Memory characteristics	a1	.77***	.04	.67	.82		.53	.18***	.31***	.17**	-.05	-.08	-.03
		b1	.66***	.12	.47	.91								
		PNDE 1	-.05	.12	-.40	.17								
		TNIE 1	.30***	.06	.20	.43	.35							
7	Appraisals	a1	.80***	.04	.73	.89		.53	.18***	.32***	.18**	-.04	-.07	-.05
		b1	.49***	.12	.20	.66								
		PNDE 1	.15	.12	-.06	.39								
		TNIE 1	.24***	.06	.10	.34	.20							
8	Memory characteristics	a1	.77***	.04	.67	.82		.52	.18***	.32***	.18**	-.04	-.07	-.05
		b1	.64***	.13	.44	.89								
		PNDE 1	-.04	.13	-.34	.17								
		TNIE 1	.29***	.06	.19	.42	.30							
9	Appraisals	a1	.54***	.09	.33	.72		.54	.18***	.31***	.20**	-.04	-.07	-.04
		b1	.58***	.14	.31	.85								
		PNDE 1	.17	.13	-.13	.45								
		TNIE 1	.19***	.06	.08	.29	.25							
	Memory characteristics	a2	.32***	.09	.12	.46								
		b2	.58***	.14	.31	.85								
		PNDE 2	-.14	.14	-.39	.13								
		TNIE 2	.11**	.05	.05	.22	.25							

Note. In models 5 and 7 path a represents the effect of the predictor (appraisals) on the mediator variable (coping strategies). In models 6 and 8 path a path a represents the effect of the predictor (memory characteristics) on the mediator variable (coping strategies). In model 9 path a represents the effect of the predictors (appraisals and memory characteristics) on the mediator variable (coping strategies). Path b in all models represents the effect of the coping strategies on psychological symptoms PGD. PNDE represents the direct effect of the predictor on psychological distress controlling for the effect of coping strategies and demographic and loss characteristics. TNIE represents the indirect effect of the predictor on the symptom variables via unhelpful coping strategies. Coefficients are standardized. Confidence intervals are 95%.

ρ is the value of the correlated residuals of the mediator and outcome resulting from unmeasured confounders necessary to result in non-significance of the indirect effect.
Covariates 1 = Gender, 2 = Child loss, 3 = Partner loss, 4 = Education, 5 = Months since death, 6 = Violent loss.

Table A5

Mediation models removing overlapping depression symptoms

									Covariates					
Model		Path	Coefficient	SE	Lower CI	Upper CI	ρ	R^2	1	2	3	4	5	6
	**Depression**							.38	.15**	.30***	.19**	-.10	-.07	-.07
10	Appraisals	a1	.80***	.04	.73	.89								
		b1	.40**	.14	.16	.65								
		PNDE 1	.09	.13	-.17	.30								
		TNIE 1	.20**	.07	.08	.33	.10							

Note. In model 10 path a represents the effect of the predictors (appraisals and memory characteristics) on the mediator variable (coping strategies). Path b in all models represents the effect of the coping strategies on psychological symptoms PGD. PNDE represents the direct effect of the predictor on psychological distress controlling for the effect of coping strategies and demographic and loss characteristics. TNIE represents the indirect effect of the predictor on the symptom variables via unhelpful coping strategies. Coefficients are standardized. Confidence intervals are 95%.

ρ is the value of the correlated residuals of the mediator and outcome resulting from unmeasured confounders necessary to result in non-significance of the indirect effect.

Covariates 1 = Gender, 2 = Child loss, 3 = Partner loss, 4 = Education, 5 = Months since death, 6 = Violent loss.
